# A protocol for a multicentre, randomised, double-blind, placebo-controlled trial to compare the effect of annual infusions of zoledronic acid to placebo on knee structural change and knee pain over 24 months in knee osteoarthritis patients – ZAP2

**DOI:** 10.1186/s12891-018-2143-2

**Published:** 2018-07-18

**Authors:** Dawn Aitken, Laura L. Laslett, Guoqi Cai, Catherine Hill, Lyn March, Anita E. Wluka, Yuanyuan Wang, Leigh Blizzard, Flavia Cicuttini, Graeme Jones

**Affiliations:** 1 Menzies Institute for Medical Research, University of Tasmania, Private Bag 23, Hobart, TAS 7000 Australia; 2 The Queen Elizabeth Hospital, University of Adelaide, Woodville, SA 5011 Australia; 3 Discipline of Medicine, University of Adelaide, Adelaide, SA 5005 Australia; 4 The University of Sydney, Royal North Shore Hospital, Sydney, NSW 2006 Australia; 5 Department of Epidemiology and Preventive Medicine, Monash University, Alfred Hospital, Melbourne, 3004 Australia

**Keywords:** Zoledronic acid, Osteoarthritis, Cartilage, Pain, Magnetic resonance imaging (MRI)

## Abstract

**Background:**

Bisphosphonates are a class of drugs that slow bone loss and are a promising candidate to treat knee osteoarthritis (OA) patients. In a pilot study, we demonstrated that zoledronic acid reduced knee pain and size of subchondral bone marrow lesions (BMLs) over 6 months in knee OA patients with significant knee pain and BMLs. A longer, larger study is required to assess whether decreases in BML size will translate to reductions in cartilage loss over time. We are currently conducting a multicentre, randomised, double-blind, placebo-controlled trial over 24 months that aims to compare the effect of annual infusions of zoledronic acid to placebo on knee structural change (assessed using magnetic resonance imaging (MRI)) and knee pain in knee OA patients.

**Methods:**

Two hundred sixty-four patients with clinical knee OA, significant knee pain and subchondral BMLs present on MRI will be recruited in Hobart, Melbourne, Sydney and Adelaide. They will be randomly allocated to the two arms of the study, receiving an annual identical intravenous infusion of either 100 mL of fluid containing zoledronic acid (5 mg/100 mL) or placebo (0.9% NaCl 100 mL), at baseline and 1 year later. MRI of the study knee will be performed at screening, month 6 and 24. Knee structure, symptoms and function will be assessed using validated methods. The primary outcome is absolute change in tibiofemoral cartilage volume (mm^3^) over 24 months. Secondary outcomes include improvement in knee pain over 3, 6, 12, 18, and 24 months and reductions in BML size over 6 and 24 months. The primary analyses will be intention-to-treat analyses of primary and secondary outcomes. Per protocol analyses will be performed as the secondary analyses.

**Discussion:**

This study will provide high-quality evidence to assess whether zoledronic acid has a novel disease modifying effect in OA by slowing cartilage loss and reducing pain. If zoledronic acid proves effective, it suggests great potential for cost savings through a delay or reduced need for joint replacement surgery, and potential for great improvements in quality of life for OA suffers.

**Trial registration:**

Australian New Zealand Clinical Trials Registry: ACTRN12613000039785, registered on 14 January 2013.

## Background

Osteoarthritis (OA) is a major cause of pain, functional limitations and disability worldwide [1], with hip and knee OA ranked as the 11th highest contributor to global disability and 38th highest in disability-adjusted life years (DALYs) [2]. Current therapies focus on alleviating pain but pain control remains poor in 50% of patients [3]. Furthermore, despite the large disease burden OA has, there are currently no approved disease-modifying OA drugs (DMOADs) that can prevent or stop the joint damage that the disease causes. Therefore, there is a major need to develop new, effective therapies.

The overall lack of treatment efficacy for OA may be partly due to treating everyone as if they have the same pathological process. OA is a complex, heterogeneous disease with multiple phenotypes [4, 5]. Treatment can be optimised by selecting study populations by subgroups with specific features that are likely to respond to particular treatments. One such phenotype is a “bone–specific phenotype” [6].

Subchondral bone marrow lesions (BMLs), visible on magnetic resonance imaging (MRI) have been shown to be an important feature in OA. On MRI they appear as regions of increased signal intensity within the bone marrow, and are a promising target for therapy. In animal models they are the first sign of OA after experimental ligament damage and precede cartilage erosion and degeneration [7]. In humans they are strongly correlated with knee pain [8–10]. Both incident [8] and progressing [10, 11] BMLs are associated with the development of knee pain. Further, a reduction in BML size is associated with pain improvement [11]. Importantly, BMLs are also associated with structural changes. They predict site-specific joint space narrowing (JSN) in those with symptomatic knee OA [12], progression of cartilage defects [13, 14] and cartilage loss on MRI [14–17]. BMLs also predict total knee replacement over periods of up to 4 years [11, 18–22]. In some studies, BMLs predict knee replacements more strongly than other predictors assessed in the same cohort [18, 21].

Bisphosphonates are a class of drugs that slow bone loss and are commonly used to treat osteoporosis. Bisphosphonates are a potential candidate for treating bony phenotypes of OA. In animal studies, bisphosphonates (alendronate, tiludronate) improve OA-related progression of structural damage [23–25]. In humans, data from general practice datasets demonstrate that bisphosphonate use (including ibandronate, pamidronate, risedronate, zoledronate) reduces risk of incident knee replacement in older women by 26% [26]. However, efficacy of bisphosphonates remains complex [27] and therefore effects on OA outcomes may differ by bisphosphonate type. While the effect of bisphosphonates on OA outcomes could be a class effect, bisphosphonates given intravenously appear to have greater treatment effects, at least, for osteoporosis [28]. A recent meta-analysis [29] of randomised controlled trials (RCTs) concluded that bisphosphonates were ineffective for reducing symptoms and radiographic progression in knee OA, but the studies included were dominated by trials of risedronate (92% of included patients received oral risedronate) [30, 31]. The authors went on to highlight that bisphosphonates may be beneficial in certain patients subgroups (e.g. patients who display high rates of subchondral turnover) [29]. Trials testing risedronate for knee OA have demonstrated reduced markers of cartilage degradation and bone resorption, but no differences in pain, radiographic joint space width (JSW), or osteophyte formation over 12–24 months compared to placebo [30, 31].

Alendronate was the most commonly used bisphosphonate in the Osteoarthritis Initiative (OAI) (> 60%) [32]. Indeed, women using bisphosphonates (i.e. alendronate) experienced reduced knee pain in the first 3 years of observation. There was a trend to less JSN in bisphosphonate users over time (year 4, 0.51 vs 0.29 mm; *p* = 0.06). There was no difference in risk of knee replacement, but the study was underpowered to assess this outcome. However, in another study, alendronate use had no effect on Western Ontario and McMasters Universities Osteoarthritis Index (WOMAC) outcomes after 6 months, and this study did not assess any structural outcomes [33]. Data from the Fracture Intervention Trial showed that alendronate retarded spinal osteophyte progression and disc space narrowing, suggesting that alendronate may have a structural effect on pathological processes in spinal OA [34].

Intra-articular (IA) clodronate (versus hyaluronic acid) was ineffective for knee pain in patients with knee OA over 5 weeks [35], but intravenous (IV) clodronate was effective for reducing pain in erosive hand OA (versus hydroxychloroquine) at 3 months in a 24-month open randomised pilot trial [36].

Bisphosphonates may work in OA primarily through their effects in the subchondal bone. BMLs identify regions of increased subchondral turnover and therefore an OA biomarker that can predict response to bisphosphonates. The earliest evidence for effects of bisphosphonates on BMLs comes from observational data showing that BMLs are less common in persons taking alendronate [37]. Risedronate (50 mg weekly) prevented an increase in BML size over 24 months [38], although this did not reach statistical significance. In a different analysis of data from the OAI, there was a trend (*p* = 0.07) towards having decreased BMLs after 12 months, in women who commenced an oral bisphosphonate (alendronate or risedronate) [39], compared to matched controls; although the size of BMLs was similar after 12 months of observation. In our randomised, double-blind, placebo-controlled pilot trial, we demonstrated that zoledronic acid (an extremely potent bisphosphonate) reduced knee pain and size of BMLs, and increased the proportion of patients improving over 6 months [40]. Therefore, more potent antiresorptives such as zoledronic acid may be efficacious for treating pain and resolving BMLs in people with OA. Based on these findings a longer, larger study is required to assess whether the decreases in BML size relating to zoledronic acid treatment will translate to reductions in cartilage loss over time. This can be hypothesised from observational studies showing both presence and severity of BMLs predict cartilage loss [14–17].

## Objective

We are conducting a multicentre, randomised, double-blind, placebo-controlled trial over 24 months to compare the effect of annual infusions of zoledronic acid to placebo on knee structural change (assessed using MRI) and knee pain in 264 patients with clinical knee OA, significant knee pain and subchondral BMLs. We hypothesise that zoledronic acid will reduce the loss of knee cartilage volume over 24 months (primary hypothesis), improve knee pain over 3, 6, 12, 18, and 24 months (secondary hypothesis) and reduce BML size over 6 and 24 months (secondary hypothesis) compared with placebo. If zoledronic acid proves effective, it will offer a novel therapeutic approach to reduce knee OA progression.

## Methods

### Study design

The Zoledronic Acid for Osteoarthritis Knee Pain (ZAP2) study is a multicentre, randomised, double-blind, placebo-controlled trial over 24 months. The trial was registered on the Australian New Zealand Clinical Trials Registry prior to recruitment, and trial reporting will be guided by the Consolidated Standards of Reporting Trials (CONSORT) Statement [41]. We aim to recruit a total of 264 patients with clinical knee OA, significant knee pain and subchondral BMLs present on MRI. Patients will be recruited via the OA Clinical Trial Network in Hobart, Melbourne, Sydney and Adelaide, using a combined strategy, including collaboration with general practitioners, rheumatologists, and orthopaedic surgeons, as well as advertising through local and social media. Patients will be encouraged to contact their local research nurse via email or telephone. Each site aims to recruit 66 patients. Ethics approval has been obtained from the Tasmania Health and Medical Human Research Ethics Committee (H0012941), The Alfred Hospital Ethics Committee (03/13), Monash University Human Research Ethics Committee (CF14/1064–2,014,000,452), Northern Sydney Local Health District Ethics Committee (HREC/13/HAWKE/80) and Human Research Ethics Committee (TQEH/LMH/MH) (HREC/13/TQEHLMH/134). Written informed consent will be obtained from all patients by the study doctor (i.e. a rheumatologist or supervised rheumatology advanced registrar).

### Inclusion criteria

The inclusion criteria are as follows: males and females with significant knee pain on most days (defined as a pain score ≥ 40 mm on a 100-mm visual analogue scale (VAS)); aged ≥50 years old; with a subchondral BML present on MRI; and meeting the American College of Rheumatology (ACR) criteria for symptomatic knee OA [42], assessed by a rheumatologist.

### Exclusion criteria

The exclusion criteria were as follows:

1. Prior use of bisphosphonates, except according to the washout schedule:

- 2 years (if use > 48 weeks).
- 1 year (if used > 8 weeks but < 48 weeks).
- 6 months (if used > 2 weeks but < 8 weeks).
- 2 months (if used < 2 weeks).
- Any intravenous bisphosphonate within the prior 2 years.

2) History of non-traumatic iritis or uveitis.
3) Abnormal blood tests [serum calcium > 2.75 mmol/L (11.0 mg/dL) or < 2.00 mmol/L (8.0 mg/dL) or creatinine clearance < 35 ml/min].
4) Serum 25-hydroxyvitamin D concentrations < 40 nmol/L. Patients with serum 25-hydroxyvitamin D concentrations < 40 nmol/L will have the option to be prescribed vitamin D supplementation and can enter the trial once their serum 25-hydroxyvitamin D concentration level is ≥40 nmol/L.
5) Use of any investigational drug(s) and/or devices within 30 days or 5 half-lives (whichever is longer) of the drug prior to randomisation.
6) Prior diagnosis of cancer (metastatic cancer or cancer diagnosed < 2 years ago where treatment is still ongoing).
7) Poor dental fitness: A dental exam with appropriate preventative dentistry will be considered prior to treatment with bisphosphonates in patients with concomitant risk factors (e.g. cancer, chemotherapy, corticosteroids, poor hygiene).
8) Severe knee OA (JSN on X-ray of Grade 3 using the Osteoarthritis Research Society International (OARSI) atlas [43]).
9) Other forms of arthritis in which disease is active and concomitant medication is used (e.g. rheumatoid arthritis or other inflammatory arthritis).
10) Patients who have undergone arthroscopy or open surgery in the index knee in the last 12 months.
11) Women who are pregnant or breast feeding.
12) Patients who have had a corticosteroid injection in the last 3 months or a hyaluronic acid injection in the last 6 months in the index knee.
13) Planned joint replacement surgery.
14) Contraindication to MRI scanning (for example, implanted pacemaker, metal sutures, presence of shrapnel or iron filings in the eye, claustrophobia, knee too large for coil).
15) Inability to give informed consent.

### Randomisation and blinding

Allocation of patients in a 1:1 ratio to either the active or placebo group will be based on computer generated random numbers using a central randomisation website hosted by the University of Tasmania. Block randomisation, using a block size of 10 (5 in each arm) will be used. This means that of every 10 patients randomised, 5 will receive active and 5 placebo. The randomisation will be stratified by study site. This will be conducted by a staff member at each study site with no direct involvement in the study.

The randomised controlled trial will be a double-blind one, with both patients and investigators assessing outcomes blinded to treatment allocation. Allocation concealment and double blinding will be ensured by 1) the use of identical IV infusions for each group; 2) objective measures of knee structural changes being made by trained observers blinded to group allocation; and 3) subjective measures being taken by research nurses blinded to group allocation.

Emergency unblinding will be allowed in limited situations that impact on the safety of study patients. Code-break for the full randomisation schedule will be maintained by the University of Tasmania. Patients who are unblinded will be withdrawn from treatment but will continue to be followed as per the planned follow-up schedule.

### Intervention

All patients will continue usual care by their treating health practitioners. Eligible patients will receive an annual identical intravenous infusion of either 100 mL of fluid containing zoledronic acid (5 mg/100 mL) or placebo (0.9% NaCl 100 mL), at baseline and 1 year later.

#### VOLT01 sub-study

During this trial, an industry funded sub-study will be added to the Hobart site. Zoledronic acid infusions are often accompanied by the side effects of acute phase reactions. This is characterised by flushing, fever, joint pains, and muscle aches in the period of time just after infusion (around 3 days post-infusion), and affects approximately 30% of patients [44]. The VOLT01 sub-study aims to examine whether adding 10 mg of methylprednisolone immediately following a zoledronic acid infusion can reduce the rate of acute phase reactions. Therefore, approximately half way through the trial the randomisation schedule will change at the Hobart site only, where patients will be randomised to one of three identical treatments: zoledronic acid, zoledronic acid PLUS methylprednisolone (VOLT01), or placebo. The details of this sub-study will be written up in a separate paper. We do not foresee that this sub-study will influence the integrity of this larger trial, which will only analyse data from patients randomised to zoledronic acid or placebo. The study rheumatologist at the Hobart site will obtain informed consent from suitable study patients for their participation in this sub-study.

### Study procedure and time points

Research nurses will first conduct screening over the telephone. If early checks of study eligibility are favourable, study patients will be booked in for a face-to-face screening visit to further determine eligibility and explain what is involved in the study. At the face-to-face visit, patients will complete questionnaires, have a knee x-ray and MRI, a blood test, and a clinical assessment by a study doctor to ensure inclusion criteria are met. The study knee will be defined as the one with symptomatic OA meeting all inclusion criteria.

Table 1 outlines the schedule of assessments. After screening, there will be 4 study visits (month 0, 6, 12 and 24). The same researcher nurses, who are blinded to treatment allocation, will measure all clinical variables, administer questionnaires, monitor compliance, and record adverse events at these visits. Additional questionnaire mail outs will occur at months 3 and 18. Infusions will occur at months 0 and 12; MRI scans will occur at screening, month 6 and 24; knee x-ray will be performed at screening; blood samples are taken at screening and 6 months, and urine samples are taken at baseline and 6 months. Three days following the infusion, patients are contacted by phone interview to assess side effects of acute phase reactions.

**Table 1:** Schedule of assessments

	Screening	Baseline (Month 0)	Day 3 Post-Infusion	Month 3	Month 6	Month 12	Day 3 Post-Infusion	Month 18	Month 24
Informed consent	x								
Clinical examination	x								
Knee x-ray	x								
Bloods	x				x*				
Knee MRI	x				x				x
Randomisation		x							
Clinic measures									
Leg strength		x			x	x			x
Height and weight		x			x				x
First void fasting urine		x*			x*				
Infusion		x				x			
Questionnaire measures									
Demographics (sex, date of birth)	x								
Knee VAS	x	x		x	x	x		x	x
Knee WOMAC		x		x	x	x		x	x
Medication use	x			x	x	x		x	x
Knee surgery	x			x	x	x		x	x
Knee joint injection					x	x		x	x
Safety (adverse events)		x	x	x	x	x	x	x	x
Acute phase reactions			x				x		
Hand VAS		x		x	x	x		x	x
Back VAS		x		x	x	x		x	x
AQoL-4D		x			x				x
Overall change in pain and function									x
Treatment guessing									x

*Only being performed at the Hobart, Melbourne and Sydney study sites

*MRI* magnetic resonance imaging, *VAS* visual analogue scale, *WOMAC* Western Ontario and McMasters Universities Osteoarthritis Index, *AQoL-4D* The Assessment of Quality of Life

### Quality assurance

To ensure high-quality execution of the trial in accordance with the protocol, all trial staff will be trained by the chief investigators and provided with a standard protocol book (with details of standard operating procedures used, trial contacts, visits, measurements, and monitoring) and case report forms.

### Outcome measures

#### Primary outcome measure: Absolute change in tibiofemoral cartilage volume (mm^3^)

Knee MRI acquisition at the four study sites is presented in Table 2, including details of sequences and parameters being used. Tibial cartilage volume will be assessed on the sagittal T1-weighted sequences by means of image processing on an independent workstation using OsiriX software (University of Geneva, Geneva, Switzerland). The volumes of tibial cartilage plates (medial tibia and lateral tibia) will be isolated from the total volume by manually drawing disarticulation contours around the cartilage boundaries on a section by section basis. These data will then be re-sampled by means of bilinear and cubic interpolation for final 3-D rendering. In our previous study, we demonstrated a coefficient of variation (CV) of 2.1% for the medial tibia and 2.2% for the lateral tibia [45], using this method.

**Table 2:** Magnetic resonance imaging sequences and parameters at the four study sites

	Machine and coil	T1-weighted sagittal	Proton density-weighted sagittal
Hobart (Note: used two different MRI scanners. Patients had their follow-up scans on the same scanner in which they had their screening scan).	1.5 T whole-body MR unit (GE Optima 450 W, Milwaukee, USA), using a dedicated 8-channel knee coil 1.5 T whole-body MR unit (Siemens, Espree), using a dedicated 15-channel knee coil	T1-weighted fat-saturated 3D gradient-recalled acquisition; flip angle 30 degrees; repetition time 38 msec; echo time 3 msec; field of view 16 cm; 512 × 512 matrix; 1 excitation; slice thickness 1.5 mm T1-weighted fat-saturated 3D gradient-recalled acquisition; flip angle 30 degrees; repetition time 31 msec; echo time 6.8 msec; field of view 16 cm; 512 × 512 matrix; 1 excitation; slice thickness 1.5 mm	Proton density fat-saturated 2D fast spin echo sequence; flip angle 150 degrees; repetition time 3800 msec; echo time 35 msec; field of view 16 cm; 512 × 512 matrix; 3 excitations; slice thickness 3 mm Proton density fat-saturated 2D fast spin echo sequence; flip angle 150 degrees; repetition time 3830 msec; echo time 39 msec; field of view 16 cm; 512 × 512 matrix; 3 excitations; slice thickness 3 mm
Melbourne	3.0 T whole-body MR unit (Philips, Achieva, Medical Systems), using a commercial 16-channel transmit receive knee coil	T1-weighted fat-saturated 3D gradient-recalled acquisition; flip angle 15 degrees; repetition time 25.9 msec; echo time 9.2 msec; field of view 16 cm; 320 × 320 matrix; slice thickness 0.5 mm	Proton density fat-saturated 2D fast spin echo sequence; flip angle 90 degrees; repetition time 3814 msec; echo time 25 msec; field of view 16 cm; 720 × 720 matrix; slice thickness 2.5 mm
Sydney	1.5 T whole-body MR unit (Siemens, Aera) using a dedicated 15-channel transmit-receive knee coil	T1-weighted fat-saturated 3D gradient-recalled acquisition; flip angle 30 degrees, repetition time 31 msec; echo time 6.8 msec; field of view 16 cm; 512 × 512 matrix; slice thickness 1.5 mm	Proton density fat-saturated 2D fast spin echo sequence; flip angle 150 degrees; repetition time 3830 msec; echo time 39 msec; field of view 16 cm; 512 × 512 matrix; slice thickness 3 mm
Adelaide	1.5 T whole-body MR unit (Siemens, Aera) using a dedicated 15-channel transmit-receive knee coil	T1-weighted fat-saturated 3D gradient-recalled acquisition; flip angle 30 degrees, repetition time 14.7 msec; echo time 6.74 msec; field of view 16 cm; 448 × 448 matrix; 1 excitation; slice thickness 1.5 mm	Proton density fat-saturated 2D fast spin echo sequence; flip angle 180 degrees; repetition time 3200 msec; echo time 39 msec; field of view 16 cm; 320 × 320 matrix; 1 excitation; slice thickness 3 mm

Knee femoral cartilage volume will be determined on the sagittal T1-weighted sequences by means of image processing on an independent workstation using Cartiscope™ (ArthroLab Inc., Montreal, Quebec, Canada), as previously described [46–48]. The segmentation of the cartilage-synovial interfaces will be carried out with the semi-automatic method under reader supervision and with corrections when needed. Cartilage volume will be evaluated directly from a standardised view of 3D cartilage geometry as the sum of elementary volumes. In our previous study, we demonstrated a CV of approximately 2% [46]. The cartilage volume assessment will be done for the medial and lateral condyles delineated by the Blumensaat’s line [48].

Tibiofemoral cartilage volume will be calculated as the sum of both the tibial and femoral compartments at screening and 24 months.

#### Secondary outcome measures

##### Improvement of knee pain at 3, 6, 12, 18, and 24 months

Knee pain will be assessed using a 100 mm VAS by asking “on this line, thinking about your *right/left* knee, where would you rate your pain, using the last 7 days as a time frame”. We will also assess pain using WOMAC [49], as we used this instrument to demonstrate a clinically significant change in BML size (140 mm^2^) [11]. Five items of WOMAC pain scale in 100-mm visual analog format [50] will be used to assess pain during walking, using stairs, in bed, sitting or lying, and standing during the last 7 days. Items will be summed to create a total WOMAC pain score (range, 0–500). Incomplete items will be addressed according to the WOMAC user guide [51]. The WOMAC pain score will be considered invalid if there is more than one missing item. In the case there is only one missing item, the remaining four items will be averaged and then multiplied by five.

##### Reduction in BML size over 6 and 24 months (mm^2^)

BMLs will be defined as an ill-defined hyperintensity in the subchondral bone, on MRI. BMLs will be assessed on the sagittal proton density weighted sequences at the medial tibial, medial femoral, lateral tibial, lateral femoral and patella sites by means of image processing on an independent workstation using OsiriX software (University of Geneva, Geneva, Switzerland). The maximum size of each lesion will be measured in mm^2^ using software cursors applied to the greatest area of the lesion, as previously described in our pilot study [40]. Previously we have demonstrated an intraclass correlation coefficient (ICC) of 0.97 [11], using this method. Total BML size (mm^2^) will be calculated as the sum of every lesion within the medial tibial, medial femoral, lateral tibial, lateral femoral and patella sites at screening, 6 and 24 months.

#### Other measurements

##### Knee function

Knee function will be assessed using WOMAC [49] at months 0, 3, 6, 12, 18 and 24. Seventeen items of the WOMAC function scale in 100-mm visual analog format [50] will be used to assess function during descending stairs, ascending stairs, rising from sitting, standing, bending to floor/picking up an object, walking on flat surface, getting in/out of the car, going shopping, putting on socks/stockings, rising from bed, taking off socks/stockings, lying in bed, getting in/out of the bath, sitting, getting on/off the toilet, heavy domestic duties, and light domestic duties during the last 7 days. Items will be summed to create a total WOMAC function score (range, 0–1700). The WOMAC function score will be considered invalid if there are more than 2 missing items. In the case there are two or less missing items, the remaining items will be averaged and then multiplied by 17 [51].

##### The assessment of quality of life (AQoL)

Health related quality of life and utility will be assessed using The Assessment of Quality of Life (AQoL-4D) questionnaire [52] at 0, 6 and 24 months.

##### Co-pathology present on MRI

Cartilage defects will be assessed at the medial tibial, medial femoral, lateral tibial, lateral femoral and patella sites as we have previously described [53]: grade 0 = normal cartilage; grade 1 = focal blistering and intracartilaginous low-signal intensity area with an intact surface and base; grade 2 = irregularities on the surface or base and loss of thickness < 50%; grade 3 = deep ulceration with loss of thickness > 50%; and grade 4 = full-thickness chondral wear with exposure of subchondral bone. In our previous study we demonstrated the ICCs ranged from 0.80–0.95 [53] for the different knee sites, using this method.

Meniscal extrusion will be assessed as we have previously described [14] as the proportion of the menisci affected by a partial or full extrusion (yes/no) at the anterior, middle, and posterior horns (medially/laterally). In our previous study we demonstrated the intra and inter-reader ICC’s ranged from 0.85–0.92 for meniscal extrusion [54].

##### Knee surgery and joint injections

Whether the patient underwent any knee surgery (including arthroscopies or joint replacement surgery) during the trial, will be assessed by questionnaire at screening, month 3, 6, 12, 18 and 24 months. Study patients will also give their consent to have their data linked to the Australian Orthopaedic Association National Joint Replacement Registry (AOANJRR). Whether the patient had a joint injection during the trial will be assessed at month 6, 12, 18 and 24 months.

##### Lower limb muscle strength

Lower limb muscle strength is a key correlate of pain and tends to increase when pain is reduced [55]. We will assess leg strength by dynamometry at the lower limb (involving both legs simultaneously) at months 0, 6, 12 and 24. The muscles measured in this technique are mainly the quadriceps and hip flexors. The previously published repeatability estimate (Cronbach’s α) for this method is 0.91 [45].

##### Overall change in pain and function

At 24 months patients will be asked to rate their overall change in pain and function (compared to baseline) on this scale: Much Worse, Moderately Worse, Slightly Worse, No Change, Slightly Better, Moderately Better, and Much Better.

##### Anthropometry

We will measure height (stadiometer), weight (electric scales) and body mass index (BMI) (weight/height^2^) at month 0, 6, and 24 months.

##### Radiographic knee OA

A standing anteroposterior semiflexed radiograph of the study knee will be performed at screening. X-rays will be scored for joint space narrowing on a four point scale (0–3) using the OARSI atlas [43]. In our hands this method has very high reproducibility with an ICC of 0.98 for joint space narrowing and 0.99 for osteophytes [56].

##### Concomitant medication

There are no restrictions with regard to concomitant analgesic medications. Medication usage (including prescription, over-the-counter, and natural/herbal remedies) will be documented at screening, month 3, 6, 12, 18 and 24 months. Patients will be asked to keep medications as stable as possible but if there are changes to the medications used or dose changes during the trial the reason will be documented.

##### Blood samples

Blood tests (Urea Electrolytes and Creatinine (UEC), calcium and vitamin D assays) will occur for safety at screening to assess inclusion criteria. Storage of blood samples will occur at screening and 6 months for future testing at the following study sites: Hobart, Melbourne, and Sydney. The blood will be stored at − 80 °C.

##### Urine samples

Storage of first void fasting urine samples will occur at month 0 and 6 for future testing at the following study sites: Hobart, Melbourne, and Sydney. The urine will be stored at − 80 °C.

##### Treatment guessing

At 24 months patients will be asked what treatment they think they received with the following options: zoledronic acid (active treatment), placebo, or not sure.

##### Other site pain

Hand pain and low back pain will be assessed at months 0, 3, 6, 12, 18 and 24 using a 100 mm VAS by asking “on this line, thinking about your *most painful hand/low back*, where would you rate your pain, using the last 30 days as a time frame”.

### Safety assessment

Adverse events will be monitored throughout the study. Standard safety and efficacy monitoring will be performed through regular face-to-face visits and phone calls between visits. The patients are requested to report any adverse events to the research staff spontaneously. Details of the adverse event and its relationship with study intervention will be recorded and reported to the Ethics Committees.

Patients will be phoned 3 days following their infusion in order to determine if they have experienced any symptoms of acute phase reactions, including flushing, fever, joint pains, and muscle aches.

### Sample size calculations

#### Primary outcome

In our pilot study [40] we found that zoledronic acid significantly reduced the size of BMLs over 6 months compared to placebo. Therefore our sample size calculations for this trial were modelled based on the assumption that a decrease in BML size will translate to a reduction in cartilage volume loss over time. This assumption is valid based on the unequivocal evidence from observation data demonstrating that BMLs predict cartilage volume loss over time [14–17, 38, 54, 57–59].

The changes in BML area seen in our pilot study were − 198.6 mm^2^ (zoledronic acid treatment) and − 22.8 mm^2^ (placebo) with regression root mean square error of 261.5 mm^2^ (i.e. standard deviation (SD)) [40], at 6 months. Unpublished observational data from our Tasmanian Older Adult Cohort (TASOAC) study allowed us to model the effect a decrease in BML size would have on tibiofemoral cartilage volume loss over 24 months. Using follow-up data for 120 TASOAC participants with BMLs, we used linear regression to estimate the relationship between absolute change in tibiofemoral cartilage volume and final BML area with adjustment for age, sex, BMI and length of follow-up. The results can be used to predict each subject’s two-year change in tibiofemoral cartilage volume from their final BML area, and the change when final BML area is reduced by 198.6 mm^2^ (expected under treatment) or 22.8 mm^2^ (expected under placebo). To take account of individual variation, the calculations were repeated in 1000 replications with the exact changes in final BML area replaced by random values of 198.6 mm^2^ (treatment) firstly and 22.8 mm^2^ (placebo) secondly, and with SD 261.5 mm^2^ in each case. The resulting estimates of tibiofemoral cartilage loss, which are expected from the 6 month changes in BML area, were − 824.0 mm^3^ (SD 273.0) in the treatment group and − 928.3 mm^3^ (SD 272.3) in the placebo group. With this difference, 132 patients (allowing for 20% drop out over 24 months) recruited to each arm of the trial will provide 80% power with 5% probability of type I error (alpha = 0.05). This estimate is conservatively based on 6-month change in BML area for *n* = 59 patients in our pilot study [40] and assumes no further improvement in BMLs after this time (although they may continue to reduce with zoledronic acid over 24 months).

#### Secondary outcome

For our secondary outcomes (improvement in pain and reduction in BML size) a sample size of 132 patients in each arm will provide 98.9 and 99.6% power to detect the adjusted treatment differences in knee pain scores and BML area respectively that were observed in our pilot study [40].

### Statistical analysis

The primary analyses will be intention-to-treat analyses of primary and secondary outcomes. Per protocol analyses will be performed as the secondary analyses.

Changes in absolute tibiofemoral cartilage volume, knee pain and BML size will be analysed using a linear mixed model with treatment, month and their interaction (treatment × month) as covariates. The correlation within trial centres and the repeated measures will be addressed using trial centre and patient identification as random intercepts. Month will be treated as random effect to allow different treatment effects among patients over time. Change in outcome measures within each group and difference of the changes between groups from baseline to follow-up will be calculated using linear combinations of the estimated coefficients. If there are baseline imbalances between treatment groups, we will consider adjusting for them based on whether we regard the imbalance as clinically significant. Missing data caused by loss to follow-up and nonresponses will be addressed by adding baseline complete variables that can explain the missingness to the regression models.

Secondary analysis for missing data will be performed using multiple imputation by chained equations. Baseline variables with complete data will be used for data imputation assuming missing at random.

Pre-specified stratified analyses will be performed to examine which subgroups may respond better to treatment. Potential stratification variables include radiographic knee OA and co-pathology present on MRI. Statistical significance will be set as a two-sided *P* value < 0.05.

### Data integrity and management

All collected data are recorded using case report forms which will be processed centrally at the Menzies Institute for Medical Research, University of Tasmania. The hard copies of the case report forms will be stored in a locked area at each study site with secured and restricted access. The electronic data will be stored on password protected servers with restricted access. All data collected will be kept strictly confidential. Daily back ups of all electronic data will occur to minimise any risk of lost data. Data transfer will be encrypted with all data de-identified. Only members of the research team who need to contact study patients, enter data or perform data quality control will have access to patient information.

After study completion, paper copies of data will be archived in secure storage. Identifiers will not be removed, in case follow-up of study patients is necessary; however, electronic data will continue to be kept in a secure electronic database. This will remain password protected and with access given only to the study investigators unless otherwise authorised by the study team.

### Withdrawal

If patients withdraw from the study before 24 months of follow-up, the reason and date will be recorded. Patients who withdraw after a minimum of 9 months will be requested to have a third MRI scan on their study knee.

### Roles and responsibilities and monitoring

The University of Tasmania (as the trial sponsor) and the principal investigators are responsible for all aspects of the trial, including design, conduct and oversight. The principal investigators will monitor the conduct and progress of the project at each site. The trial coordinator will visit each study site to make sure that all trial procedures are compliant with the trial protocol. The principal investigators and the research team will have regular teleconferences to ensure efficient study execution and ongoing monitoring of the study progress, with summary documents circulated after each meeting. A Data and Safety Monitoring Board was not convened for this trial as zoledronic acid is approved in Australia by the Therapeutic Goods Administration (TGA) and has a well-known safety profile. The trial is also being monitored at each site by a practicing rheumatologist with experience prescribing zoledronic acid.

### Dissemination plans

The results of this study will be presented at conferences and published in scientific journals. Any notes or publications arising from our research will be de-identified. Only aggregate statistical results will be presented.

The outcomes of the project will be disseminated to study patients using non-technical language. The scientific paper will be available for dissemination to patients’ should they wish to receive it, after the manuscript has been accepted for publication. Dissemination of the overall study findings to the patients will occur in a de-identified manner and be based on the entire study population.

## Discussion

We proposed a multicentre, randomised, double blind placebo controlled trial to determine whether annual infusions of zoledronic acid reduces the rate of knee cartilage volume loss, improves knee pain and reduces BML size, compared to placebo in people with clinical knee OA, significant knee pain and subchondral BMLs. If zoledronic acid proves effective, it will offer a novel therapeutic approach to reduce knee OA progression.

Zoledronic acid is an established treatment for osteoporosis [28, 60]. Bisphosphonates have effects through a variety of mechanisms, including effects on the subchondral bone and osteochondral junction [6]. Bisphosphonates may also have anti-inflammatory actions [61, 62]; which may play a role in an immediate analgesic benefit, as distinct from that which might arise as a consequence of osteochondral structural alteration, and thus may explain why analgesic benefits may not persist beyond the period of drug use. Overall, the mechanism and direction of effect remains controversial, the evidence suggests that bisphosphonates have effects on the subchondral bone.

Radiographs are a tool commonly used to assess disease progression in OA, but it is not the optimal method. It is moderately responsive to change in terms of standardised response means (SRM) [63]; however, it is insensitive to change in cartilage measures [64]. MRI offers a much better assessment, and OA features on MRI are better targeted for defining and following disease progression. Using MRI, cartilage volume/thickness loss predict knee replacement [18, 65–67] and have similar levels of sensitivity to discriminate treatments in clinical trials [38]. Accordingly, MRI assessment of cartilage morphology is now recommended for the evaluation of disease progression as an endpoint for clinical trials [66, 68]. Simultaneously, we will assess change in knee pain over time using a 100 mm VAS, and WOMAC [49] as secondary endpoints. Thus, the findings from this study will show whether zoledronic acid treatment has both symptom modifying and disease modifying effects.

Zoledronic acid is associated a suite of well characterised acute phase reactions [44]. While these reactions are of mild-moderate intensity, and self-limiting in duration, they are common (incidence ~ 30%) and unpleasant. We included a sub-study to investigate the efficacy of adding 10 mg methylprednisolone immediately following the zoledronic acid infusion. Methylprednisolone, an anti-inflammatory drug, might reduce rates of acute phase reactions by reducing inflammation due to the zoledronic acid infusion, as intermediates in the mevalonate pathway activated by blocking farnesyl pyrophosphate synthase (isopentenyl diphosphate and dimethylallyl diphosphate) accumulate in monocytes when the enzyme is blocked and result in activation of adjacent γδT cells, with the release of interferon-δ and TNF [69]. We hypothesised that administration of methylprednisolone after zoledronic acid infusion will reduce rates of acute phase reactions.

## Conclusion

In summary, knee OA is a major but poorly understood public health problem. Our novel preliminary data showed that zoledronic acid improved knee pain and reduced the size on BMLs in OA patients [40]. If zoledronic acid can reduce knee pain, size of BMLs, and slow cartilage loss, it suggests great potential for cost savings through a delay or reduced need for joint replacement surgery, and potential for great improvements in quality of life for OA suffers. The success of this study will provide scientific evidence for using a cost-effective and innovative approach to addressing this clinically significant problem and lends itself to incorporation in routine clinical practice.
